# Effectiveness of Interstitial Laser Acupuncture Depends upon Dosage: Experimental Results from Electrocardiographic and Electrocorticographic Recordings

**DOI:** 10.1155/2013/934783

**Published:** 2013-11-20

**Authors:** Wei He, Gerhard Litscher, Xiang-Hong Jing, Hong Shi, Xiao-Yu Wang, Ingrid Gaischek, Yang-Shuai Su, Daniela Litscher, Zhao-Kun Yang, Juan-Juan Xin, Ling Hu

**Affiliations:** ^1^Department of Meridians, Institute of Acupuncture and Moxibustion, China Academy of Chinese Medical Sciences, No. 16 Nanxiaojie, Dongzhimen, Beijing 100700, China; ^2^Stronach Research Unit for Complementary and Integrative Laser Medicine, Research Unit of Biomedical Engineering in Anesthesia and Intensive Care Medicine, TCM Research Center Graz, Medical University of Graz, Auenbruggerplatz 29, 8036 Graz, Austria

## Abstract

The purpose of this study was to evaluate the influence of the duration of interstitial laser acupuncture therapy effects on neurovegetative and neurobioelectrical parameters like heart rate (HR), heart rate variability (HRV), and electroencephalogram (EEG). We investigated 6 male Sprague-Dawley rats. They underwent 10 min, 20 min, and 30 min interstitial laser acupuncture (in randomized order, with a break of at least 30 min between the different measurement conditions) at the acupoint Neiguan. HR changed significantly only during 20 min red laser stimulation, whereas 10 and 30 min stimulation did not induce significant changes. HRV did not change significantly during any of the different durations; however, an increase was found during 20 min irradiation. Neither the LF/HF ratio of HRV nor the integrated EEG showed significant changes. In this study, it could be experimentally proved that some effects of laser acupuncture are time dependent, and therefore the dosage, as well known from theory, also depends on the time factor. We could especially demonstrate that different treatment times lead to different effects on neurovegetative and neurobioelectrical parameters. Further studies are needed to verify or refute these results.

## 1. Introduction

In a previous study, our research team from China and Austria investigated interstitial (i.st.) laser acupuncture in anesthetized Sprague-Dawley rats for the first time [1]. In that preliminary study, we explored i.st. laser acupuncture, intravenous laser blood irradiation, and electroacupuncture under stable conditions and analyzed the effects on physiological neurovegetative parameters and bioelectrical brain activity. We found changes in the rat model; however, the question of the adequate dosage of laser is still an open one in scientific literature [2, 3].

Tunér and Hode, both are very renowned researchers on laser therapy, stated in 2010 [4]: “Anyone who studies the literature carefully can become confused. Some wavelengths achieve the best effects on this and that, while others have poorer effects or none at all. Some doses lead to beneficial effects, but when the dose is increased, the effects wear off. If we treat a condition, some of the parameters we want to influence may be affected, but perhaps not all. If we administer treatment from a distance, we do not get the same effects as if we treat in contact or with pressure. Some frequencies produce effects on pain, others on oedema. What are we to believe? And what do we do to find the best dose, wavelength, and so forth?” [4].

The goal of the present study was to change the dosage of the laser acupuncture treatment via the duration of the i.st. irradiation at the acupoint Neiguan in order to find an optimal treatment time [2]. The data were recorded in Beijing, China, and the analysis was performed in Graz, Austria.

## 2. Animals and Methods

### 2.1. Sprague-Dawley Rats

Six male healthy Sprague-Dawley rats (weight: 190–300 g) were kept in an animal house maintained at 24 ± 1°C, with a 12-hour light-dark cycle and free access to food and water for seven days before the experiment. The procedure was the same as in our previous work published recently [1]. The animals were initially anesthetized with an intraperitoneal injection of 10% urethane (1.2 g/kg, Sigma-Aldrich, St. Louis, MO, USA). Additional sodium pentobarbital was administered if necessary to prolong the anesthetic state. Animals were sacrificed by an overdose of anesthetics after the study. The study was approved by the Institutional Animal Care and Use Committee of the China Academy of Chinese Medical Sciences and was in accordance with the National Institutes of Health guidelines. 

### 2.2. Interstitial Laser Acupuncture

**Figure 1:**
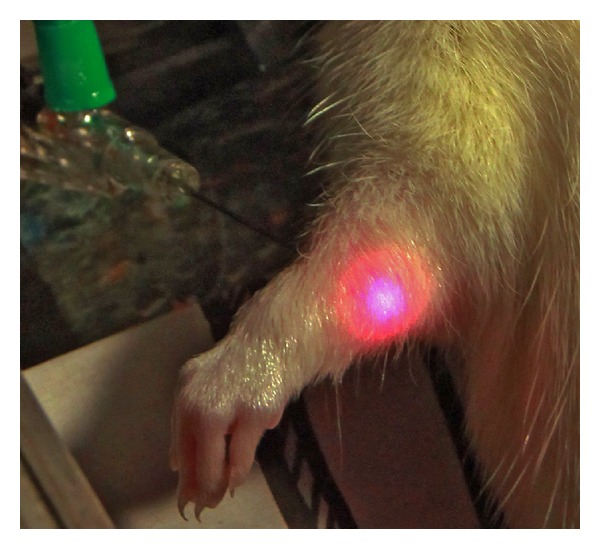
Interstitial laser acupuncture in a rat model.

The laser needle for i.st. laser irradiation (length: 35 mm; diameter: 0.55 mm) was a Modulas needle (type: IN-Light, Schwa-Medico, Ehringshausen, Germany). It emits red laser light in continuous wave mode with a wavelength of 658 nm and an output power of 50 mW (Figure 1). We stimulated the acupoint Neiguan (PC6) on the left side using i.st. laser acupuncture. The laser needle was inserted about 3 mm in the acupoint Neiguan. This acupoint is located proximal to the accessory carpal pad of the forelimb, between the flexor carpi radialis and palmaris longus ligaments [1].

### 2.3. Procedure

**Figure 2:**
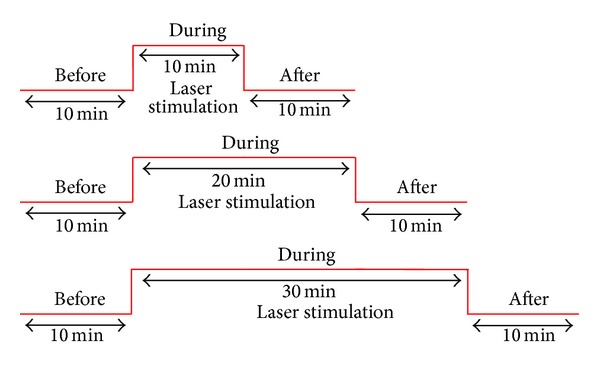
Experimental procedure for the different durations of i.st. laser acupuncture stimulation.

Figure 2 shows the measurement profile. Three measurement periods were compared: one before, one during, and one after stimulation. This scheme was used for all three conditions (10, 20, and 30 min i.st. laser acupuncture) in the same rat. The order of the stimulation methods was randomized, and the time between the separate measurement conditions was at least 30 min.

### 2.4. Measurement Parameters

As in the previous study [1], we registered electrocardiographic (ECG) and electroencephalographic (EEG) parameters using a biophysical amplifier AVB-10 (Nihon Kohden, Japan). For the ECG, we evaluated heart rate (HR), heart rate variability (HRV), and the LF (low frequency)/HF (high frequency) ratio of HRV. Again, EEG was registered directly on the brain; high cutoff frequency was 100 Hz, and the low cutoff frequency was 0.5 Hz.

### 2.5. Statistical Analysis

The data were analyzed using Friedman repeated measures analysis of variance (ANOVA) on ranks (SigmaPlot 12.0, Systat Software Inc., Chicago, IL, USA). Post hoc analysis was performed using Holm-Sidak test. The level of significance was defined as *P* < 0.05.

## 3. Results

**Figure 3:**
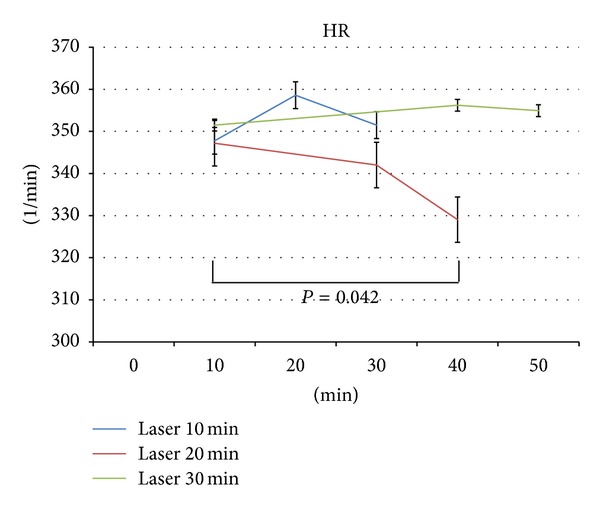
Mean heart rate (HR) of the 6 rats. Note the different stimulation durations (10, 20, and 30 min). Significant changes were only found for a duration of 20 min (red line). The error bars indicate the standard error (SE).

The analysis of the HR of all 6 rats is shown in Figure 3. Note the significant (*P* = 0.042) decrease of HR after 20 min i.st. laser acupuncture stimulation at the left Neiguan acupoint. It is also interesting that stimulation durations of 10 and 30 min, respectively, did not lead to the same effects in the rat model.

**Figure 4:**
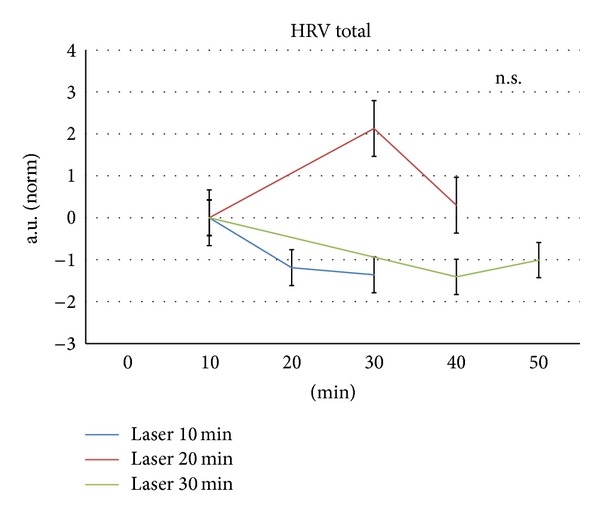
Changes in total heart rate variability (HRV total) before, during, and after the three stimulation procedures. a.u. (norm): normative arbitrary units. For further explanation, compare with Figure 3.

In contrast to HR, total HRV increased (insignificantly), also during 20 min laser stimulation. No increases in total HRV were seen during or after 10 min or 30 min laser stimulation (Figure 4).

**Figure 5:**
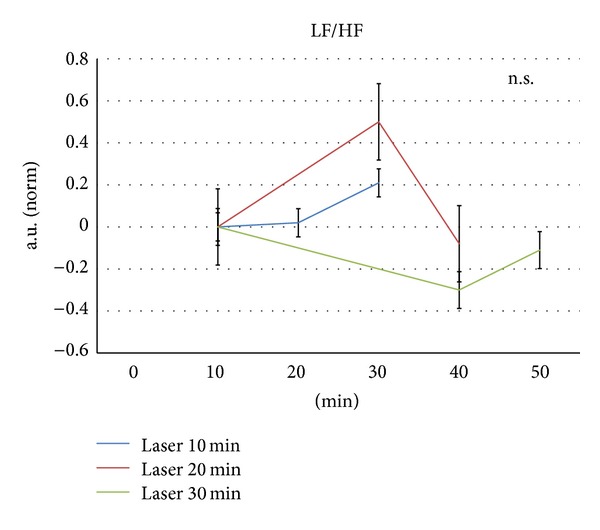
LF/HF of the 6 investigated rats. For further explanation, see Figures 3 and 4.

Figure 5 shows the changes of LF/HF HRV ratio. No significant changes were found during any of the stimulation procedures.

**Figure 6:**
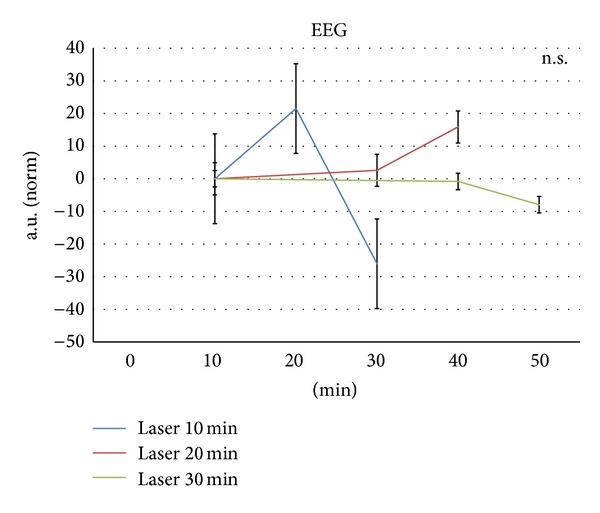
Integrated electrical rat brain activity. Note the (insignificant) increase of the integrated EEG after 20 min laser stimulation. For further explanation, see Figures 3 and 4.

Analysis of the bioelectrical brain activity (EEG, Figure 6) did not reach the level of statistical significance. 

## 4. Discussion

Interstitial laser acupuncture is a new acupuncture modality that also allows treatment of different body areas like spinal nerves or joints. The application of laser energy can be performed directly in the region of interest.

In our first animal experimental study, we could demonstrate that there are significant changes in neurovegetative parameters like HR and HRV after i.st. laser stimulation of the left Neiguan acupoint in rats [1]. In a human pilot study, it could also be shown that the pain intensity of patients with shoulder pain and spine-associated pain could be significantly reduced [5]. Interstitial laser therapy was also already used as a therapy for liver metastases in Western medicine. First results of a clinical phase I study were published already 10 years ago by a German research team [6]. The authors of that study stated that i.st. laser therapy of liver malignancies is a minimally invasive procedure with little side effects which produces sharply defined, yet small volumes of necrosis [6].

In the last years, there was a continuous increase in publications concerning laser acupuncture (see http://www.pubmed.gov/). However, there are still some important questions concerning dosage and in this context especially treatment duration.

The term treatment dose is identical to energy density, which is measured in watt seconds per cm² (= joule (J) per cm²). Dosage refers to the amount of energy per unit area brought to bear on tissue or cell culture [4].

As we have shown in many previous studies, some people can feel the laser, others not [7]. Maybe it is appropriate to begin with a low dose for a new patient in the first treatment session to be sure that one does not enter a biosuppressive dose range [4]. In laser acupuncture, the dose is often given in joules per point. It is assumed that a “point” is something small. Tunér and Hode [4] have defined an “acupuncture point” as an area that is 5 mm in diameter (~0.2 cm²) or less. They stated that “this means if we hit the skin with the light concentrated to this small area and administer 1 J “per point,” we have given 1 J “per point,” and in this “point” (~0.2 cm²) the dose value is 5 J/cm²” [4]. The authors of the book [4] further stated that the most common situation in laser therapy is the wish to administer a certain dose (*D*) to a specified area (*A*) with a laser, having an (average) output power (*P*), and therefore it is necessary to calculate the treatment time (*t*) for the laser probe at hand. If the problem to treat is situated at a depth (*d*) (where *d* = 0 to 4 cm; this is the maximum penetration depth of the red laser [2, 8]), the following approximate formula can be used to find the treatment time:

(1) $$\begin{array}{c} t=\frac{D\times A}{P}\times \left(1+d\right)\;\left[\text{sec}\right]. \end{array}$$

For this formula to work, the correct units to be used are as follows: *P* must be given in watts (not milliwatts); *D* must be given in J/cm²; *A* must be expressed in cm²; and *d* in cm. The treatment time will then come out in seconds.

If we use this formula for our laser acupuncture experiment in rats, the calculated time for effects seen in laser acupuncture is too small (*t* = {(5 × 0.2)/0.04}×(1 + 1) = 25 sec). Even if we insert *d* = 4 cm (humans), the estimated treatment time (*t* ≈ 2 min) seems to be too short. Our measurements within this study only showed significant effects on neurovegetative parameters in rats during or after the 20 min stimulation duration.

Although the therapeutic use of laser acupuncture in general is gaining popularity, objective evaluation of the dosage-dependent effects is very difficult [9]. Only few studies describe the important parameters like wavelength, irradiance, and the beam profile in detail. For a complete description of the dosage-dependent effects, energy transmission factors have also to be taken into account. These factors are, for example, skin properties such as thickness or pigmentation [9]. The thickness of the skin starts decreasing at the age of 45 years, and the difference in pigmentation between the Caucasian and, for example, African population, which is caused by different concentrations of melanin [10], leads to different penetration depths of the laser beam.

Our present study is the first one comparing different i.st. laser stimulation treatment times (durations) in rats. It is well known that the effectiveness of laser acupuncture depends upon dosage [3, 4]. We used a red laser (658 nm) with an output power of about 40 to 50 mW, which results in a very high dosage. This dosage is also time dependent. In our study with the experimental rat model, we could demonstrate that different treatment times lead to different effects on neurovegetative and neurobioelectrical parameters. Further studies are needed to verify or refute these results.
